# Genetic Modifications That Expand Oncolytic Virus Potency

**DOI:** 10.3389/fmolb.2022.831091

**Published:** 2022-01-26

**Authors:** Francisca Cristi, Tomás Gutiérrez, Mary M. Hitt, Maya Shmulevitz

**Affiliations:** ^1^ Shmulevitz Laboratory, Department of Medical Microbiology and Immunology, Faculty of Medicine and Dentistry, University of Alberta, Edmonton, AB, Canada; ^2^ Goping Laboratory, Department of Biochemistry, Faculty of Medicine and Dentistry, University of Alberta, Edmonton, AB, Canada; ^3^ Hitt Laboratory, Department of Oncology, Faculty of Medicine and Dentistry, University of Alberta, Edmonton, AB, Canada

**Keywords:** oncolytic virus, cancer therapy, genetic modifications, oncolytic potency, metastasis

## Abstract

Oncolytic viruses (OVs) are a promising type of cancer therapy since they selectively replicate in tumor cells without damaging healthy cells. Many oncolytic viruses have progressed to human clinical trials, however, their performance as monotherapy has not been as successful as expected. Importantly, recent literature suggests that the oncolytic potential of these viruses can be further increased by genetically modifying the viruses. In this review, we describe genetic modifications to OVs that improve their ability to kill tumor cells directly, to dismantle the tumor microenvironment, or to alter tumor cell signaling and enhance anti-tumor immunity. These advances are particularly important to increase virus spread and reduce metastasis, as demonstrated in animal models. Since metastasis is the principal cause of mortality in cancer patients, having OVs designed to target metastases could transform cancer therapy. The genetic alterations reported to date are only the beginning of all possible improvements to OVs. Modifications described here could be combined together, targeting multiple processes, or with other non-viral therapies with potential to provide a strong and lasting anti-tumor response in cancer patients.

## Introduction

Since first observing that viruses can induce tumor regressions a century ago (Bierman et al., 1953; Bluming and Ziegler, 1971), the possibility of using viruses as a cancer therapy has maintained the interest of many scientists. Oncolytic viruses (OVs) selectively target tumor cells, leaving healthy cells unharmed. Their mechanisms are multi-dimensional; OVs infect and lyse tumor cells, generating local tumor burden reduction (Kooti et al., 2021; Santos Apolonio et al., 2021). Some OVs also infect and kill tumor-supporting cells in the tumor microenvironment such as endothelial cells and fibroblasts (Pikor et al., 2015), helping dismantle the environment that supports the tumor. Finally, many OVs induce cytokine secretion and expose tumor-associated antigens, which favours an anti-tumor immune response (Lichty et al., 2014). The struggle faced by scientists is to enhance the potency of these three activities to a point where the cancer is fully eliminated. Several OVs have progressed to human trials and even achieved FDA approval for specialized use in patients (Fukuhara et al., 2016; Cook and Chauhan, 2020; Macedo et al., 2020). For example, the herpes viruses T-VEC, G207 and G47Δ (Rider et al., 2019; Uche et al., 2021); the adenovirus DNX-2401 or Tasadenoturev (Ene et al., 2021) and Oncorine (Liang, 2018); the reovirus pelareopep (Müller et al., 2020); the vaccinia virus Olvi-Vec (Manyam et al., 2021); and the coxsackievirus CAVATAK (Annels et al., 2019). However, many articles and reviews have reiterated that OVs developed thus far are insufficient as a monotherapy. Accordingly, two major strategies are being explored to boost the cancer therapeutic potency of OVs; one is to combine OVs with other cancer therapies, and the second is to modify OVs further genetically to increase their potency (Zainutdinov et al., 2019; Santos Apolonio et al., 2021). This review will focus on the latter; genetic modifications to OVs that produce improvement in their ability to kill tumor cells directly, dismantle the tumor microenvironment, or promote anti-tumor immunity. In the future, combining genetically modified OVs with other OVs and/or other non-viral therapies, may present a feasible path to using viruses in cancer therapy.

While this review focuses on genetic strategies to make OVs more potent, it is important to recognize that OVs must first exhibit strong tumor selectivity and safety. OVs include RNA viruses such as reovirus, Newcastle disease virus (NDV), vesicular stomatitis virus (VSV), measles virus (MV), poliovirus and coxsackievirus; and DNA viruses such as parvovirus, adenovirus, vaccinia virus and herpes simplex virus (HSV). Some of these OVs are inherently more infectious towards transformed cells, such as reovirus (Strong et al., 1998; Norman et al., 2004; Shmulevitz et al., 2005) and parvovirus (Nüesch et al., 2012; Geiss et al., 2017). These viruses are naturally cleared by healthy tissues, but features of tumors such as specific cell receptors, intracellular enzymes, or reduced antiviral response, favour replication of these viruses in tumor cells. Other viruses need to be genetically modified to be more selective towards tumor cells, such as adenovirus, vaccinia virus or HSV. For example, a common strategy to make adenoviruses selective to tumor cells is genetic manipulation of the essential adenovirus E1A protein (Niemann and Kühnel, 2017). E1A is necessary for adenoviruses to replicate in non-transformed cells because one of its functions is to inactivate cellular retinoblastoma protein and activate transcription factor E2F-induced cell cycle genes (Niemann and Kühnel, 2017; Sohn and Hearing, 2019). The retinoblastoma binding activity of E1A is dispensable for replication in tumor cells that already harbor dysregulated cell cycle and compensate for the absence of this E1A activity (Fueyo et al., 2000). A different approach is to selectively transcribe indispensable viral proteins under the control of specific transcription factors that are upregulated in tumors; for example, placing E1A gene expression under the control of the hTERT promoter (Shay and Bacchetti, 1997; Wirth et al., 2003). Additional modes of selectivity will be described for specific OVs throughout the review, as a prelude to novel genetic modification strategies that increase *potency*.

Development of secondary tumors at sites distal to the primary tumor (metastasis) is one of the main challenges in cancer therapy and the principal cause of mortality (Fares et al., 2020). Metastasis is a complex process that involves a cascade of steps, starting with activation of invasion and metastatic phenotype (Hanahan and Weinberg, 2011). Epithelial cells undergo a process of *trans*-differentiation known as epithelial-mesenchymal transition (EMT) to acquire the ability to migrate, invade, resist stress, and disseminate (Lambert et al., 2017). Cancer cells are then able to spread from the local tumor environment to intravasate blood and lymphatic vessels. Cancer cells travel through the lymphatic and blood systems as single cells or in clusters. Ultimately, these cells arrest and extravasate through endothelial cells to colonize secondary sites, where they can proliferate immediately or stay in a dormant state for even years depending on environmental factors (Hanahan and Weinberg, 2011; Lambert et al., 2017; Fares et al., 2020). OVs with their tumor selectivity and the potential to be delivered systemically are a promising therapy against metastasis. This review will therefore describe genetic modifications that increase OV potency in general, but also focus on strategies that directly aim to enhance potency against metastases.

## Engineering OVS to Enhance Virus Replication and Killing in the Primary Tumor and Metastatic Sites

One of the ways by which OVs exert their oncolytic activities is by directly infecting and killing cancer cells. One strategy to increase oncolytic potency is therefore to increase the OV’s replication or tumor killing abilities. Enhanced OV replication in tumors would also amplify secondary effects, such as increases in tumor antigen presentation, anti-tumor immune cell recruitment, and virus dose for dissemination to distal sites of metastasis. Keeping in mind that no known natural virus requires tumors as host, but rather that researchers have harnessed viruses for this task, it is not surprising that viruses need to be genetically modified or selected to be optimally infectious and lethal towards cancer cells. The question becomes, what specific changes are needed to make a given virus thrive better in targeted cancers? Below are examples of diverse and sometimes unpredictable changes to OVs that promote direct infection and killing activities. These are summarized in Table 1.

**TABLE 1:** Oncolytic viruses with enhanced virus replication and killing ability.

Virus backbone	New virus	Modification	Reason	Result	Reference
HSV^a^	Synco-2D	Addition of hyperfusogenic glycoprotein of gibbon ape leukemia virus into Baco1	Increase fusogenic ability	Increased survival and reduced metastasis in mouse ovarian, prostate and breast cancer	(Nakamori et al., 2003, 2004a, 2004b)
HSV	OncSyn	gBsyn3 syncytial mutation incorporated into an already attenuated HSV-1 virus (NV1020)	Increase fusogenic ability	Reduced mouse tumor growth and metastasis breast cancer model	(Israyelyan et al., 2007, 2008)
HSV	OncdSyn	A second syncytia-enhancing mutation introduced into viral glyco-protein K of OncSyn	Increase fusogenic ability	Reduced and/or inhibited mouse breast tumor metastases	Israyelyan et al. (2008)
HSV	ΔN146	Truncation of the viral protein γ_1_34.5 (to aa 147–263) rather than deletion as in most oncolytic HSV	Keep some anti-viral subversion functions	Reduced mouse breast tumor growth and lung metastases	Liu and He, (2019)
Adenovirus	Ad5-Δ24RGD	Addition of RGD (Arg-Gly-Asp) to fiber	Expand receptor recognition	Prolonged survival in a mouse metastatic breast tumor model	Ranki et al. (2007)
Adenovirus	ColoAd1 or Enadenotuvirev	Directed evolution	Enhance killing ability	Reduced mouse colon cancer metastasis to liver	Kuhn et al. (2008)
Adenovirus	TelomeKiller	Addition of the red fluorescent protein KillerRed with E1A and E1B driven by the hTERT promoter	Generation of reactive oxygen species (ROS) upon green light irradiation	Reduced lymph node metastases size in a mouse rectal tumor model	Takehara et al. (2016)
VSV^b^	rVSV-NDV/FL (L289A))	Addition of a Newcastle disease virus fusion protein	Increase fusogenic ability	Prolonged survival in a multifocal liver metastases rat model	(Ebert et al., 2004; Yamaki et al., 2013)
VSV	VSV-p14	Addition of p14 fusion protein from reptilian repovirus	Increase fusogenic ability	Reduced tumor growth and increased survival in mouse breast and colon cancer models	Le Boeuf et al. (2017)
VSV	VSV-GP	Addition of the lymphocytic choriomeningitis virus (LCMV) glycoprotein	Increase safety and reduce neuro-toxicity	Prolonged survival and reduced tumor growth in a mouse melanoma model	(Muik et al., 2011, 2014; Kimpel et al., 2018)
Reovirus	T3v1, T3v2	Directed evolution	Increase replication	Prolonged survival in a mouse melanoma model	(Shmulevitz et al., 2012; Mohamed et al., 2015a)
Adenovirus		Overexpression of adenovirus death protein (ADP)	Early cell death	Tumor growth reduction	Doronin et al. (2000)

a HSV, herpes simplex virus.

b VSV, vesicular stomatitis virus.

### Herpes Simplex Virus

Herpes simplex virus, type 1 (HSV-1) is an enveloped, double-stranded linear DNA virus (Watanabe and Goshima, 2018). HSV-1 has a large genome, encoding at least 83 genes that function to mediate virus replication, modulate the host cell, and subvert the immune response. As is commonly known, HSV-1 replication in mucosa causes cold sores, but HSV-1 can also persist latently in trigeminal ganglia, sometimes causing encephalitis upon reactivation. When transforming HSV-1 into an OV, it is important to eliminate neuro-invasive abilities to reduce the risk of encephalitis (Kanai et al., 2012). Two virus genes commonly deleted from HSV-1 to destroy neural tropism are UL56 and γ34.5. The UL56 protein associates with host kinesin motor protein KIF1A to facilitate neuroinvasion (Koshizuka et al., 2005). The γ34.5-encoded ICP34.5 protein blocks cellular protein translation and anti-viral responses and is necessary for virus replication in neurons. For example, G207 is a second generation genetically modified HSV-1 with deletions in γ34.5 and an inactivating insertion of *LacZ* in the *ICP6* gene. The latter encodes the large subunit of the viral ribonucleotide reductase, a key enzyme for DNA synthesis. The combination of both deletions permits replication in tumor cells while preventing a productive infection in normal tissue (Mineta et al., 1995; Uche et al., 2021). Patient-derived xenograft studies in nude mice showed that pediatric brain tumors are particularly sensitive to G207 (Friedman et al., 2018). Accordingly, G207 was recently tested in a phase 1 trial in pediatric malignant high-grade glioma. Patients showed an increase in tumor infiltrating CD3^+^, CD4^+^ and CD8^+^ lymphocytes and no serious adverse effects related to G207 administration. Median overall survival was 12.2 months, in contrast with the 5.6 median overall survival usually observed in this setting (Friedman et al., 2021). G47Δ is a third generation HSV-1 based on G207 with an additional deletion in the *α47* gene, involved in antigen presentation. G47Δ is more effective than G207 at preventing tumor growth in animal models, while showing a similar safety level (Todo et al., 2001). G47Δ received the Orphan Drug Designation and the conditional approval for the treatment of malignant glioma in Japan.

Another important HSV-1 for oncolytic therapy is Talimogene Laherparepvec (T-VEC). This virus has deletions in the γ34.5 gene as well as the gene encoding ICP47 involved in suppressing antigen presentation. Furthermore, the human granulocyte macrophage colony-stimulating factor (GM-CSF) cDNA was incorporated into T-VEC to increase recruitment and activation of antigen-presenting cells to tumors (Conry et al., 2018). The combination of safety and immunomodulation has made T-VEC an effective therapy against melanoma (Liu et al., 2003). In 2015, after successful phase I, II and III clinical trials, T-VEC was approved by the U.S. Food and Drug Administration (FDA) and European Medicines Agency for clinical use (Andtbacka et al., 2015; Mondal et al., 2020). The approval of this engineered oncolytic virus, T-VEC, is a great advancement to the use of OVs in patients (Mondal et al., 2020).

Although T-VEC improved median overall survival from 18.9 to 23.2 months in clinical trials of 436 patients with unresectable stage IIIB to IV melanoma (Andtbacka et al., 2015), there remains interest to further augment T-VEC potency towards melanoma patients. Moreover, research is still necessary to enhance potency of HSV-derived OVs towards an assortment of other cancer types. Given that HSV-1 depends on membrane fusion for entry, assembly and cell-cell spread (Weed and Nicola, 2017), two independent research groups focused on increasing potency by augmenting the fusogenic ability of HSV-derived OVs. The Zhang group designed a new version of a fusogenic HSV (called Synco-2D), in which the hyperfusogenic glycoprotein of gibbon ape leukemia virus was incorporated into the already fusogenic “Baco 1” mutant HSV (Nakamori et al., 2003). In a xenograft mouse model of peritoneal metastatic ovarian cancer established by Hey-8 cells, Synco-2D exhibited a dramatic effect on tumor growth and mouse survival (8/8 survivors) relative to Baco-1 virus (5/8 survivors) and PBS (0/8 survivors). The same group demonstrated that intravenous injection of Synco-2D, reduced metastases in both the PC-3M-Pro4 prostate cancer xenograft model and the 4T1 cell metastatic syngeneic breast tumor model (Nakamori et al., 2004a; Nakamori et al., 2004b). Interestingly, they found that Synco-2D caused increased CD8^+^ T cell activation and antitumor immunity in the 4T1 model. Also, since results suggest that Synco-2D is a promising oncolytic virus that targets metastasis in three different animal tumor models, it would be important to compare Synco-2D directly to T-VEC and other competing HSV-based OVs in clinical testing.

The Kousoulas group (Israyelyan et al., 2007) also created a new fusogenic HSV-based OV called “OncSyn.” OncSyn was built upon the ‘NV1020’ attenuated HSV-1 containing modifications that eliminate UL56, α0, γ34.5, and α4 to confer safety and tumor selectivity. Into NV1020, the authors introduced a single amino acid change (the gBsyn3 mutation) in the surface viral fusion protein, glycoprotein B, resulting in bigger syncytial plaques and increased virus replication. OncSyn was efficient *in vivo* at reducing tumor growth in a xenograft mouse model system using MDA-MB-435S-lux human breast cancer cells. Later, a second syncytia-enhancing mutation was introduced into the viral transmembrane glycoprotein K, creating the “OncdSyn” OV (Israyelyan et al., 2008), enhancing fusion of otherwise resistant cells. Both OncSyn and OncdSyn were tested in the highly metastatic interscapular 4T1 breast tumor model. Both viruses reduced the number of mice with metastases in internal organs such as liver, spleen, and kidneys, with OncdSyn exhibiting slightly better response at early timepoints than OncSyn. However, OncSyn and OncdSyn were not compared with the parental virus NV1020 in the *in vivo* setting, and hence it is not possible to conclusively attribute an advantage to the fusogenic mutants.

Enhancing HSV-derived OVs replication in tumors can also be achieved by fine-tuning the precise modification of HSV-1 genes such as γ_1_34.5. As mentioned, the γ_1_34.5 HSV-1 gene blocks cellular IFN-mediated anti-viral response, but also activates protein translation by inhibiting phosphorylation of translation initiation factor eIF2α. The γ_1_34.5 gene is deleted from most HSV-based OVs such as T-VEC, which contributes to specificity of these OVs towards cancer cells that often harbor compromised IFN signalling. The He group (Liu and He, 2019) questioned whether removing the N-terminal domain of γ34.5 responsible for IFN-mediated antiviral subversion was sufficient for specificity, and whether keeping the remaining domains of γ34.5 that facilitate activation of protein translation could promote OV replication in tumors. In comparison to the γ34.5 null mutant (Δ γ34.5), an N-terminal truncation mutant of γ34.5 (ΔN146) achieved higher viral replication in tumor cells *in vitro* and was more resistant to IFN- α exposure. In the metastatic 4T1 syngeneic breast tumor mouse model, ΔN146 treatment significantly reduced lung metastases relative to Δ γ34.5 and PBS treatments (10 vs. 15 vs. 25 nodules/lung). These studies suggest that the ΔN146 mutant, which maintains ability to stimulate virus protein synthesis, may provide a better oncolytic virus *in vivo* to reduce metastasis.

### Adenovirus

Adenovirus has a broad tissue tropism, so for safety reasons oncolytic adenoviruses must be modified to selectively replicate in tumor cells. Once specificity is achieved, adenovirus can be further modified to encode exogenous proteins that favour virus replication/production in tumors. The natural receptor of adenovirus 5, Coxsackievirus–adenovirus receptor, is not abundant in cancers; so, one approach to increase the infectivity of this virus in cancers is to expand receptor recognition. In Ranki et al. (2007), the RGD (Arg-Gly-Asp) domain was added to the fiber protein encoded by a recombinant adenovirus with a truncated E1A gene (Ad5-Δ24), creating the Ad5-Δ24RGD. RGD is a ligand for αβ integrin receptors which are abundantly expressed in malignant cancer cells (Wu et al., 2019). Ad5-Δ24RGD significantly prolonged survival when compared with an Ad lacking the RGD domain (Ad5Δ24E3) in the M4A4-LM3 xenograft metastatic breast cancer model. Strangely, no differences in the presence of primary tumors, as monitored by fluorescence emission of GFP positive M4A4-LM3 cells, were observed in mice treated with the two viruses. More detailed animal studies are required to understand how binding to αβ integrins promoted survival in this metastatic model. Interestingly, the Ad5-Δ24RGD virus has been tested and improved during the last years, creating what is currently commercially known as DNX-2401 or Tasadenoturev. This modified adenovirus received an Orphan Drug Designation by the FDA for recurrent glioblastoma (Philbrick and Adamson, 2019; Ene et al., 2021).

As a complement to rationally engineering OVs to thrive in cancer cells as described above, directed evolution has provided a worthwhile strategy to enhance OV replication in cancer cells. In Kuhn et al. (2008), pools of adenovirus serotypes were passaged on human tumor cell lines to promote recombination and emergence of more-potent viral variants. The ColoAd1 variant, also called Enadenotucirev, was exhibited higher oncolytic activities in colon cancer cell cultures. ColoAd1 is derived from the Ad11p serotype, which is less prevalent in the human population than Ad5. Since most patients are seronegative for Ad11p, ColoAd1 may be less-quickly neutralized by host antibodies providing an advantage in systemic administration for metastatic cancers, although an advantage of host seroprevalence for ColoAd1 has yet to be empirically demonstrated. When evaluated in a metastatic colon cancer xenograft mouse model, ColoAd1 reduced growth of liver metastases relative to the parental Ad11p virus. ColoAd1 also replicated better in tumor biopsies than the parental virus.

Both directed evolution and rational design approaches have demonstrated that adenovirus OV potency can be enhanced by intensifying the cell killing activities of this virus. OVs kill the tumor cells they infect during the late stages of their replication cycle. Despite that adenovirus is ultimately lytic towards tumor cells, studies found that inducing earlier cell death can promote oncolytic activity. For example, Doronin et al. (2000) found that an E1A-modified adenovirus reengineered to overexpress the adenovirus death protein (ADP) exhibited increased replication and cell-cell spread in human A549 lung carcinoma cells and reduced the size of A549-derived tumors in nude mice, relative to their parental strain. Remarkably, Uil et al. (2011) also discovered that increased expression of ADP enhances oncolytic activity of adenovirus but using an unbiased directed evolution approach. Specifically, Ad5 expressing an error-prone polymerase was used to select mutants with improved replication in SKOV-3 ovarian carcinoma cells. The variant “F421Y” was found to carry a mutation at the splice acceptor site of an ADP-encoding exon that enhanced ADP expression levels. F421Y increased cell killing of SKOV-3, human breast (SKBR-3) and prostate (PC-3) carcinoma cell lines. To our knowledge, however, these viruses have not yet been compared in immune competent animal models, nor evaluated for their activity towards metastases.

Finally, Takehara et al. (2016) took a very innovative approach to enhance cell killing by adenovirus. The authors developed TelomeKiller, a tumor-specific replicating adenovirus that expresses the red fluorescent protein KillerRed under the control of the CMV promoter inserted into E3 gene of adenovirus. KillerRed is a photosensitizer that generates reactive oxygen species (ROS) upon green light irradiation/photodynamic therapy (PDT). TelomeKiller was evaluated in an intraperitoneal lymph node metastasis model, where HCT116-GFP human colorectal cells were implanted into the submucosal layer of the rectum. Virus was directly injected into the rectal tumors. Three days later, GFP-expressing metastases were subjected to PDT. Twenty-one days after virus injection, the authors observed that the GFP signal had decreased in metastatic lymph nodes in all PDT + TelomeKiller-treated mice, whereas the signal increased in control mice or mice treated with virus but not PDT. These results suggest that TelomeKiller in combination with PDT efficiently targets lymph node metastases and that adenovirus replication alone is not enough to shrink metastases.

### Vesicular Stomatitis Virus

Vesicular Stomatitis Virus (VSV) is best known to farmers since it causes mild fever and blisters in cattle (Buijs et al., 2015). It is an enveloped single-stranded negative sense RNA virus in the family *Rhabdoviridae*. In humans, VSV selectively replicates in cancer cells which tend to have defective or reduced type I IFN responses. VSV has some advantages as an OV, including its rapid replication, lack of pre-existing immunity in humans, broad tropism, and easily manipulated genome (Simovic et al., 2015). However, in some animal studies, wild-type VSV treatment has presented neurological symptoms. Because of this, current VSV mutants are generated with mutations in their matrix protein (M) or membrane fusion protein (G) to eliminate neurotropism (Buijs et al., 2015).

Several attempts have been made to enhance VSV OV potency by increasing membrane fusion, similar to above-described modifications to HSV-based OVs. In Yamaki et al. (2013), a previously designed recombinant VSV virus (rVSV-NDV/FL (L289A)) expressing a Newcastle disease virus fusion protein was tested in two metastatic models of colorectal cancer (Ebert et al., 2004). First, when RCN-H4 colorectal cancer cells (CRC) are injected into the liver, they produce lesions in the liver. In this CRC liver metastasis model, rVSV-NDV/FL (L289A) significantly increased long-term survival. In the second model, CRCs are instead injected systemically *via* venous infusion; herein, rats develop CRC metastatic lesions in their lungs. In the systemically-administered CRC lung metastasis model, the efficiency of rVSV-NDV/FL (L289A) was less impressive, significantly prolonging survival but not generating long-term surviving rats. While survival data suggested a promising improvement to OV potency, it would have been informative to also assess the metastatic burden directly in the animal experiments. More recently, Le Boeuf et al. (2017) pseudo-typed VSV with the p14 fusion protein of fusogenic reptilian reovirus and found significant improvement of oncolytic potency in several animal models. While the VSV G protein only induces cell fusion at low pH in lysosomes, the p14 reovirus fusion protein induces membrane fusion at neutral pH. Accordingly, the VSV-p14 displayed syncytia at normal pH, and promoted higher virus yields and dissemination in cancer cell cultures and spheroids. VSV-p14 resulted in smaller tumor volumes and increased survival in the 4T1 orthotopic metastatic breast tumor model, without altering biodistribution and safety of the virus. Two additional mouse models were applied to assess if p14 incorporation into VSV improves protection against metastatic disease. First, 4T1 mammary tumors were allowed to metastasize prior to excision of the primary tumor and OV administration through the tail vein. In this model, VSV-p14 extended survival significantly more than the control VSV-GFP virus. Second, mice were intravenously administered CT26 colon cancer cells expressing lacZ then systemically treated by the OVs. In this experiment, VSV-p14 significantly reduced lacZ + metastatic nodules relative to VSV-GFP or untreated mice. Moreover, VSV-p14 seemed to increase tumor immunity; for example, increasing the number of activated CD4^+^ and CD8^+^ T cells in the spleen, draining lymph nodes, and tumors, relative to controls. With multiple independent researchers finding a benefit for syncytium formation in oncolytic potency of VSV, this seems a promising avenue to continue building upon.

Lastly, Muik et al. (2014) and Kimpel et al. (2018) found that VSV pseudo-typed with the less-immunogenic lymphocytic choriomeningitis virus (LCMV) glycoprotein (VSV/LCMV-GP) lacked VSV’s neurotoxicity, induced fewer neutralizing antibodies, and reduced lung metastasis in a syngeneic B16-OVA melanoma model (Muik et al., 2011). Specifically, mice injected with B16-OVA cells intravenously were treated with tail vein injections of VSV/LCMV-GP or left untreated. After 14 days, the number of lung metastases was significantly reduced in VSV/LCMV-GP treated mice compared to untreated mice, and the remaining metastases were smaller. While the efficiency of VSV/LCMV-GP was not compared to VSV in the B16 melanoma model to demonstrate the direct advantage of adding the LCMV-GP into VSV, previous comparisons in non-metastatic glioma xenograft models found an advantage of VSV/LCMV-GP over VSV. Regardless, the idea of using a surface glycoprotein that is less immunogenic and therefore enables multi-dosing of OVs with reduced virus neutralization is worthy of note for future developments.

### Reovirus

Mammalian orthoreovirus (reovirus) naturally circulates among humans and other mammals through the fecal-oral route without causing disease. Remarkably, unmodified serotype 3 reovirus (T3wt) was also found to infect, disseminate amongst, and kill tumor cells. Healthy untransformed cells do not support rampant replication and spread of reovirus because they have fewer enzymes that support reovirus uncoating during entry, they do not efficiently undergo cell death to release progeny virions, and because untransformed cells mount a strong interferon antiviral response. Several clinical trials have demonstrated the safety of T3wt (also commercially known as pelareopep by Oncolytics Biotech Inc.) in cancer patients, and some trials have demonstrated a moderate but improvable oncolytic effect (Clements et al., 2014). Indeed, FDA granted Orphan Drug Designation to T3wt for breast, ovarian, pancreatic, peritoneal and gastric cancers (Müller et al., 2020). In the Shmulevitz laboratory, we have isolated reovirus mutants that replicate better than T3wt in a panel of tumor cells while retaining limited replication in untransformed cells. Two of these mutants, T3v1 and T3v2, have an advantage in virus disassembly which leads to increased virus replication and larger plaque size (Mohamed et al., 2015a). In a syngeneic mouse B16 metastatic melanoma model, flank tumors were injected intratumorally with these mutants at days 14, 16 and 18 post-injection of B16 cells. T3v1 and T3v2 increased survival relative to T3wt in this metastatic model (Shmulevitz et al., 2012). We also found that genetic variations in wild-type reovirus strains impact tremendously their replication ability in different tumor cell lines such as mouse ID8 ovarian cancer, human Huh 7.5 hepatocarcinoma, human H1299 non-small cell lung carcinoma and mouse B16-F10 melanoma cell lines (Mohamed et al., 2020a). The oncolytic effects *in vitro* translated into a reduction in melanoma tumor growth in the B16 animal tumor model (Mohamed et al., 2020b). This evidence suggests that genetic modifications can improve reovirus potency in pre-clinical models which is auspicious for clinical trials. Several additional reovirus mutants have been found to promote binding, uncoating or antiviral response *in vitro* (Mohamed et al., 2015b), some of which exhibit different cell receptor tropisms (van den Wollenberg et al., 2012). Unfortunately, these mutants have not been tested in tumor models, so their oncolytic potential remains to be characterized.

## Overcoming the Extracellular Matrix Barrier to Virus Dissemination to Metastatic Sites

Metastases present a large challenge when treating late-stage cancer patients. OVs offer potential to target metastases directly, or indirectly through OV-induced anti-tumor responses. To improve direct targeting of metastases, specific strategies that promote OV dissemination have been investigated (summarized in Table 2). One such strategy involves altering the extracellular matrix to improve virus dissemination out of the local tumor and into secondary tumor sites.

**TABLE 2:** Oncolytic viruses that dismantle the tumor microenvironment to improve virus dissemination.

Virus backbone	New virus	Modification	Reason	Result	Reference
Adenovirus	Ad-ΔE1B-RLX	Addition of relaxin to Ad-ΔE1B	Improve virus distribution in the tumor	Reduced lung metastasis in a mouse melanoma model	Kim et al. (2006)
Adenovirus	OV-RLX-5T35H	Addition of relaxin to Ad5/35 adenovirus	Improve virus distribution in the tumor	Reduced metastasis and improved survival in a mouse pancreatic tumor model	Ganesh et al. (2007)
VV^a^	GLV-1h255	Addition of MMP-9 to GLV-1H68	Improve virus distribution in the tumor	Did not alter metastasis in a mouse prostate cancer model	Schäfer et al. (2012)
Adenovirus	VCN-01	Addition of hyaluronidase (PH20) into fiber-modified AdΔ24E1A	Improve virus distribution in the tumor	Reduced metastasis in a metastatic osteosarcoma mouse model	Martínez-Vélez et al. (2016)
Adenovirus	EnAdDNAse	Addition of exonuclease DNAse I into ColoAd1 Enadenotuvirev	Improve virus distribution in the tumor	Reduced tumor growth and improved virus spread	Tedcastle et al. (2016)
Adenovirus	EnAdPH20	Addition of hyaluronidase into ColoAd1 Enadenotuvirev	Improve virus distribution in the tumor	Reduced tumor growth and improved virus spread	Tedcastle et al. (2016)
Reovirus	S1-T241I	Mutation of viral binding protein sigma1 to impede cleavage by tumor proteases	Improve virus distribution in the tumor	Improved virus distribution in primary tumors	Fernandes et al. (2019)

a VV, vaccinia virus.

The tumor microenvironment consists of cells embedded in a non-cellular component, mainly extracellular matrix (ECM). The cellular component includes cancer cells, immune cells, fibroblasts, pericytes, endothelial cells, adipocytes, and mesenchymal stem cells. The spaces between the cells are composed of interstitial fluid, cell-free DNA, exosomes, as well as ECM (Baghban et al., 2020). While the composition of the tumor ECM depends on the type of tumor, the most common molecules expressed by solid tumors are fibrillar collagens, fibronectin, elastin, and laminins (Henke et al., 2019). These molecules are produced either by the cancer cells or other cells of the tumor microenvironment such as fibroblasts (Naba et al., 2012). Cancer-associated fibroblasts (CAFs) are described as great secretors of collagen, which is linked with resistance to therapies and poor prognosis (Provenzano et al., 2008; Mammoto et al., 2013). ECM in the tumor does not have the same characteristics as that in normal tissues. In the tumor, ECM is more rigid, abundant and dense (Henke et al., 2019). Because of this, tumor ECM acts as a barrier for therapeutic agents such as OVs. At the same time, the barrier impairs the oxygen and nutrients supply, activating apoptosis and senescence. ECM interactions also can lead to activation of signalling pathways in tumor cells that promote survival and avoid cell cycle arrest. In addition, tumor ECM has an important role regulating EMT and metastasis (Henke et al., 2019). Thus, the ECM is a candidate cancer therapeutic target.

At the onset of metastasis, during the invasion process, remodeling of the ECM is mainly done by metalloproteases (MMPs). MMPs are proteolytic enzymes that degrade most ECM molecules and regulate the activity of other important proteins in the tumor microenvironment such as growth factors, cytokines, chemokines, proteinases and cell receptors (Egeblad and Werb, 2002). MMPs are secreted by different cells within the tumor including cancer cells, CAFs, and neutrophils (Kessenbrock et al., 2010). MMPs are overexpressed in most cancers and are indicative of increased tumor aggressiveness and shortened patient survival (Egeblad and Werb, 2002; Hadler-Olsen et al., 2013). Given the prevalence of MMPs in tumor ECM, both adenovirus- and vaccinia- based OVs have been genetically modified to exploit the natural functions of MMPs and enhance virus dissemination.

In addition to E1A-deleted adenoviruses described in previous sections, adenoviruses lacking E1B proteins (Ad-ΔE1B) show specificity towards transformed cells and have extensively been evaluated for cancer therapy. The E1B proteins normally block p53 tumor suppressor activities and promote nuclear export of viral mRNAs (O’Shea et al., 2004; O’Shea et al., 2005); Ad-ΔE1B therefore depends on transformed cells to overcome the absence of E1B functions. To improve the distribution of E1B-deleted adenovirus, the Yun group created an adenovirus expressing relaxin (Ad-ΔE1B-RLX). Relaxin is a 6 kDa protein hormone that upregulates matrix metalloproteinases (MMPs) (Unemori et al., 1996), which in turn help degrade the ECM (Stamenkovic, 2003). The authors hypothesized that ECM impedes virus spread, and therefore that removing the ECM with relaxin would promote virus dissemination and broaden activity to metastatic sites. In Kim et al. (2006), the intratumor injection of Ad-ΔE1B-RLX in a murine syngeneic B16 metastatic melanoma model reduced tumor metastasis in lungs significantly relative to the adenovirus without relaxin (Ad-ΔE1B). While clearly the Ad-ΔE1B-RLX provided advantage over Ad-ΔE1B, the precise mechanism was not confirmed by quantifying the levels of MMPs and extent of ECM degradation. In addition, relaxin has pleiotropic activities that extend from cell signaling activation and nitric oxide production to expression of MMPs, stromal cell-derived factor (SDF)1-α and vascular endothelial growth factor (VEGF) that impact vasculogenesis (Ng et al., 2018). Each of these functional consequences could affect activity and dissemination of an oncolytic virus. Interestingly, even in ECM-devoid cell cultures, Kim et al. (2006) observed that Ad-ΔE1B-RLX has advantage over Ad-ΔE1B in establishing virus plaques more rapidly and inducing apoptosis of tumor cells. Therefore, it is likely that effects of relaxin are multifaceted, and it would be interesting to know which effects are most critical for enhancing activities of oncolytic adenovirus.

In further support for potential benefits of modifying oncolytic viruses to encode relaxin, the Jooss group also observed an advantage of adding relaxin to adenovirus; they used the Ad5/35 chimeric fiber-encoding adenovirus which exhibits tumor specificity by requiring CD46 receptors abundant on tumor cells for attachment. In the PC-3luc prostate metastatic xenograft model, the Ad5/35 chimeric adenovirus expressing relaxin (OV-RLX-5T35H) increased virus titers in the primary tumors and reduced collagen staining compared with tumors treated with the virus without relaxin (OV-5T35H) (Ganesh et al., 2007). The reduced collagen staining adds direct evidence for the effects of relaxin on the ECM. When metastases in lymph nodes and lungs were analyzed in this same tumor model, they observed that the percentage of mice with metastases was reduced to zero in the OV-RLX-5T35H-treated group from 27% in the OV-5T35H-treated group and 80% in the PBS-treated group. The reduction in metastasis correlated with increased animal survival, supporting that an engineered adenovirus expressing relaxin increases infectivity in the primary tumor, reduces metastases and improves survival.

To directly address the impact of MMPs on oncolytic virus activities, Schäfer et al. (2012) incorporated matrix metalloproteinase 9 (MMP-9) into the oncolytic vaccinia virus strain GLV-1h68, creating the new strain GLV-1h255. Tumor specificity of the original GLV-1H68 virus, also known as GL-ONC1, is conferred by removal of viral genes (specifically, F14.5L, thymidine kinase J2R and hemagglutinin A56R) and consequential dependence on tumor associated cellular processes. The addition of MMP-9 to GLV-1H68 did not change virus infectivity *in vitro* but improved tumor regression and virus titers in tumors in the PC-3 xenograft tumor model of prostate cancer. MMP-9 expression and collagen reduction were validated in primary PC-3 tumors. Intriguingly, when volumes of lumbar and renal lymph node metastases were evaluated, there were no differences between the GLV-1h68- and GLV-1H255-treated mice, suggesting that the addition of MMP-9 did not alter the metastasis-reducing effect of GLV-1h68 or, alternatively, that the increased virus mobilization was counterbalanced by the increased mobility of the tumor cells. It would be interesting to see the effects of GLV-1H255 relative to GLV-1h68 in a tumor model that differs in ECM composition or response to MMP-9. Moreover, as with all viruses discussed in this review, it would be highly informative to compare relaxin to MMP-9 in the same oncolytic virus and model system and establish if these genetic modifications produce overlapping or distinct contributions to oncolytic mechanisms.

As an alternative strategy to increase tissue permeability, Martínez-Vélez et al. (2016) introduced hyaluronidase into an oncolytic adenovirus with intentions to hydrolyze the ECM constituent hyaluronan. This adenovirus strain VCN-01 contains modifications to the viral genome that produce a more specific and powerful virus (E2F-binding motif in the E1A promoter, a modified fiber and a 24-bp deletion in the E1A gene). Moreover, VCN-01 also encodes the human PH20 gene that encodes soluble hyaluronidase and a modified fiber protein designed to increase virus half-life in blood. When administered systemically, VCN-01 reduced lung metastases by 20% in a lung metastatic osteosarcoma xenograft model (human 531 MII osteosarcoma cells injected through tail vein) relative to PBS control. It would have been worthwhile however to evaluate the contribution of each individual change in VCN-01 on reducing metastases, by comparing adenoviruses with these single and combined genomic modifications. Such studies would help suggest which of the four modifications are worth inclusion in both adenovirus and other virus-based oncolytic therapies.

Non-apoptotic cell death produced within tumors releases large fragments of DNA to the ECM (Kroemer et al., 2013; Tedcastle et al., 2016), which could also impede OV dissemination. In Tedcastle et al. (Tedcastle et al., 2016), the authors inserted the gene encoding the exonuclease DNAse I into the oncolytic adenovirus Enadenotucirev [EnAd, previously described in section A1 (Kuhn et al., 2008)], to eliminate free DNA and enhance virus spread. They also included a EnAd-based virus armed with hyaluronidase as a control (EnAdPH20). Viruses were intratumorally injected at a relative low dose [1 × 10^9^ viral particles (vp)/tumor] to be able to observe the increase in virus spread. In the DLD human colon carcinoma xenograft model, EnAdDNAse and EnAdPH20 viruses significantly inhibited tumor growth relative to PBS or unmodified virus. Virus replication and spread in the tumors 32 days post infection was higher with EnAdDNAse than with EnAd or EnAdPH20. Also, at 32 days post infection, tumors conserved enzymatic activity suggesting a persistent expression of the virus-encoded enzymes. This research exemplifies the value of comparing two different engineering approaches to increase virus spread in the same platform, since it clearly suggests the advantage of DNAse as an additional modification.

With respect to genetically modifying OVs to express ECM-degrading enzymes as a strategy to increase OV dissemination, one should consider the impact of such enzymes on tumor progression as well. ECM and MMPs play many roles in promoting cancer development; they regulate tumor growth, apoptosis, angiogenesis, invasion, metastasis as well as the anti-tumor immune response (Egeblad and Werb, 2002). In fact, inhibition of MMPs is the basis of several anticancer therapies (Winer et al., 2018). Accordingly, it is critical to ask whether expressing MMPs in OVs as a strategy to enhance OV dissemination would also come with negative consequences to cancer development. The balance between increasing virus dissemination versus inadvertently increasing cancer progression can be very difficult to achieve, and we look forward to future studies that also evaluate cancer parameters such as angiogenesis and cancer cell invasion as possible unwanted consequences of expressing ECM-degrading enzymes.

While the studies described above focused mostly on the ECM as a physical barrier to virus dissemination, our laboratory wondered if ECM might also directly impact the infectious activity of an oncolytic virus. As described in section A1, reovirus (T3wt) shows inherent specificity towards tumors with limited replication in healthy tissues (Duncan et al., 1978; Coffey et al., 1998; Norman et al., 2004). Reovirus naturally infects through the enteric tract, but infections are rapidly cleared by the immune system with little-to-no symptom (Organ and Rubin, 1998). In the enteric tract, reovirus exploits gut proteases to augment infection, so in Fernandes et al. (2019), we wondered what effect, if any, breast tumor proteases could have on reovirus infectivity. We discovered that breast tumor extracts decreased reovirus infectivity by 100-fold by cleaving reovirus cell attachment proteins and decreasing attachment of reovirus particles to breast tumor cells. Specifically, a zinc-dependent metalloprotease released by breast cancer cells was responsible for the inactivation of reovirus. To overcome this restriction, we created a reovirus with a single mutation in the protease cleavage site of the reovirus cell-attachment protein σ1 (T249I); this mutant retained attachment to breast tumor cells despite MMP presence. Future studies are necessary to determine if the T249I mutation in reovirus, by overcoming negative effects of MMPs, also promotes oncolytic activities in models of cancer metastasis. Importantly however, in contrast to the strategies described above for adenovirus- and vaccinia- based OVs that increase MMP activities to promote OV dissemination, our findings with reovirus beckon a consideration for decreasing MMP activities as a strategy to increase activities of OVs that are negatively impacted by such host enzymes. In other words, a precise understanding of the direct relationship between a specific OV and ECM-modifying enzymes in tumors seems necessary to make the most beneficial genetic modifications to OVs.

## Engineering OVS to Reduce Tumor Burden by Altering Angiogenesis

The genetic modifications described in section A focus on enhancing the replication, killing, and dissemination of OVs, so that OVs might exhibit increased direct oncolytic activities in tumors and secondary sites of metastasis. But during their habitation in tumors, OVs have the potential to also deliver exogenous genes that indirectly contribute to cancer treatment. In this section, we will discuss OVs (summarized in Table 3) genetically modified to express factors that modulate angiogenesis.

**TABLE 3:** Oncolytic viruses that inhibit angiogenesis and alter tumor signaling.

Virus backbone	New virus	Modification	Reason	Result	Reference
VV^a^	OVV-CXCR4-A-mFc	Addition of the N terminal region of CXCL2 that functions as CXCR4 antagonist	To block CXCR4 and stop cancer development	Reduced metastasis in mouse breast and ovarian tumor models	(Gil et al., 2013, 2014)
HSV^b^	34.5ENVE	Addition of Vasculostatin-120	To inhibit tumor vascular-ization	Prolonged survival and reduced metastasis in ovarian and breast tumor mouse models	(Bolyard et al., 2014; Meisen et al., 2015)
Sendai virus	rSeV/dMFct14 (uPA2) or BioKnife	Fusion protein modified to be cleaved by uPA and not trypsin	Selective killing in uPA-expressing cells	Reduced tumor burden in a mesothelioma mouse model cancer and reduced secondary tumor growth in a head and neck carcinoma model	(Morodomi et al., 2012; Tanaka et al., 2019)
Adenovirus	Ad.sTβRFc	Addition of a soluble form of TGF-β receptor II fused with human immunoglobulin Fc fragment	Inhibition of TGF-β signaling	Decreased bone metastasis and prolonged survival in mouse and prostate breast cancer bone metastatic tumor models	(Hu et al., 2010, 2011, 2012; Zhang et al., 2012)
Adenovirus	Ad.dcn	Addition of human decorin	Activation of anti-tumorigenic signaling pathways	Reduced tumor progression, prolonged survival and decreased bone and lung metastasis in breast cancer bone metastatic models	(Xu et al., 2015; Yang et al., 2015; Zhao et al., 2019)
Adenovirus	rAd.DCN.GM	Addition of human decorin and granulocyte macrophage colony stimulating factor (GM-CSF)	Activation of anti-tumorigenic signaling pathways and immune system (natural killer cells, macrophages and dendritic cells)	Reduced tumor growth and pulmonary metastasis in a mouse colorectal cancer	Liu et al. (2017)
Adenovirus	Ad5/3-D24-hTNFa	Addition of human TNFα	Activation of apoptosis	Reduced tumor growth in a xenograft mouse model of prostate cancer and a metastatic mouse melanoma model	Hirvinen et al. (2015)
Adenovirus	Ad5/3-E2F-d24-hTNFa-IRES-hIL2	Addition of human TNFα and human IL-2	Activation of apoptosis and induction of anti-tumor immunity	Reduced tumor growth in a Syrian hamster model	Havunen et al. (2017)
Adenovirus	Ad.IR-E1A/TRAIL	Addition of TNF-related apoptosis-inducing ligand (TRAIL)	Activation of apoptosis	Reduced colorectal metastases in the liver in a mouse model	Sova et al. (2004)
Adenovirus	P55-HTERT-HRE-TRAIL	Addition of TRAIL to virus with E1A controlled by the hTERT promoter and E1B controlled by a hypoxia response element	Increase tumor specificity of virus replication and apoptosis activation	Prolonged survival and decreased metastasis in a mouse metastatic breast tumor model	Zhu et al. (2013)
Adenovirus	Ad/TRAIL-E1	Addition of TRAIL, with TRAIL and E1A under the control of the hTERT promoter	Increase tumor specificity of virus replication and apoptosis activation	Reduced metastasis in a peritoneal dissemination mouse tumor model; increased apoptosis in the metastases	Zhou et al. (2017)
Adenovirus	M4	Addition of a fragment of antisense STAT3 to the backbone adenovirus Ad5/dE1A	Silencing of transcription factor STAT3	Decreased tumor growth, invasiveness, and peritoneal dissemination in an orthotopic mouse model of gastric cancer	Han et al. (2009)
Adenovirus	ZD55-SATB1	Addition of SATB1 shRNA	Silencing of transcription factor SATB1	Decreased primary tumor growth and inhibited pulmonary metastasis in a metastatic prostate cancer model	Mao et al. (2015)
VV^a^	OVV-BECN1	Addition of Beclin-1	Activation of autophagy	Reduced tumor growth in xenograft murine models of leukemia and non-Hodgkin lymphoma	(Lei et al., 2020; Xie et al., 2021)
NDV^c^	rNDV-18HL	Addition of an antibody against CD147	Blocking of CD147	Reduced liver metastasis and prolonged survival in an orthotopic mouse hepatoma model	Wei et al. (2015)
Adenovirus	Ad.wnt-E1A (delta24bp)-TSLC1	Addition of TLSC1; Viral protein E1A expression under the control of Wnt promoter	Cancer stem cell specificity of virus replication and increasing expression of TLSC1	Reduced liver metastasis in a hepatocellular carcinoma mouse model	Zhang et al. (2017)

a VV, vaccinia virus.

b HSV, herpes simplex virus.

c NDV, newcastle disease virus.

Endothelial cells (ECs) are main components of blood vessels and important elements of the tumor microenvironment because they supply the nutrients and oxygen requirements of growing tumors. Angiogenesis, the process of creating new blood vessels, is therefore fundamental for cancer development and a common target for cancer therapy (Hanahan and Folkman, 1996; Bergers and Benjamin, 2003; Potente et al., 2011; Mander and Finnie, 2018). Angiogenesis is regulated by soluble factors such as the C-X-C chemokine ligand 12 (CXCL12) and its receptor type 4 (CXCR4) (Guo et al., 2016; Najafi et al., 2019). While CXCL12 is mainly secreted by cells associated with the tumor microenvironment, CXCR4 is expressed by ECs, cancer cells and cancer stem cells (Cornelison et al., 2018; Yi et al., 2019). CXCL12/CXCR4 signaling promotes an immunosuppressive environment, ECM remodeling, reprogramming of tumor cells, tumor angiogenesis, and metastasis (Mortezaee, 2020). In particular, the role of CXCL12/CXCR4 in angiogenesis is well described. ECs in the tumor microenvironment overexpress CXCR4 in response to hypoxia (Schioppa et al., 2003) and the secretion of CXCL12 by tumor cells and cells in the tumor microenvironment, recruits ECs into the tumor (Salcedo and Oppenheim, 2003). CXCL12 secretion also influences the transformation of tumor cells to mimic blood vessels (Yang et al., 2016). Importantly, the blocking of CXCL12/CXCR4 axis inhibits tumor growth and impairs metastasis (Sun et al., 2013; Zhou et al., 2019a).

CXCR4 blocking has been evaluated in several clinical trials as a strategy to reduce cancer development, but since this chemokine is abundantly expressed at both tumor and non-tumor sites, CXCR4 blockade specifically in tumors can be difficult. To resolve the specificity issue, the Kozbor group in Gil et al. (2013) designed a tumor-selective vaccinia virus expressing the N terminal region of CXCL12 that functions as a CXCR4 antagonist (OVV-CXCR4-A-mFc). Specifically, they used the vaccinia Western Reserve strain with thymidine kinase (TK) and vaccinia growth factor (VGF) genes interrupted to make the virus tumor specific. They incorporated either EGFP or CXCR4-A-mFc into the TK locus. The efficacy of the viruses to target metastasis was then evaluated in the syngeneic mouse 4T1 breast tumor model. 4T1 cells were orthotopically implanted and when cells were disseminated to the lungs, virus was injected intravenously. Histologic analysis showed that the control group had an average of 20 metastatic nodules in the lungs, whereas OVV-EGFP- and OVV-CXCR4-A-mFc-treated animals had 6.6 and 2.6 metastatic colonies, respectively. They also evaluated the efficacy of the OVV-CXCR4-A-mFc when it was administered before or after excision of the primary tumor. For the pre-operative setting, mice were injected with 4T1 cells then 10 days later, virus was injected. Primary tumors were resected 8 days after virus injection. The OVV-CXCR4-A-mFc-treated group showed higher survival compared with control and OVV-EGFP. In the post-operative setting, tumors were resected 18 days after cell injection and then viruses were injected. In this experiment, OVV-CXCR4-A-mFc-treated mice survived longer than control and OVV-EGFP-treated mice. More importantly, survival for OVV-CXCR4-A-mFc group was longer in the post-operative setting than the pre-operative setting (42% vs. 20% disease-free after 110 days). These studies suggest that injecting viruses after tumor resection was more efficient at targeting metastases, and that the addition of the CXCR4 agonist promoted the oncolytic activities of vaccinia virus. The Kozbor group observed similar results in a syngeneic metastatic model of ovarian cancer (ID8-T cells, which are derived from ascites of ID8 tumor-bearing mice) (Gil et al., 2014). They associated the increased survival following OVV-CXCR4-A-mFc treatment with a reduction of CXCL12 and VEGF as well as cancer-initiating, endothelial, myeloid and plasmacytoid dendritic cells in the tumor microenvironment. They also detected increased activated T cell infiltration and anti-tumor immune response.

In addition to chemokines, there are many factors that control vascularization of specific tissues. Brain-specific angiogenesis inhibitor 1 (BAI1) is an orphan G protein-coupled receptor that is cleaved extracellularly to release a 120 kDa fragment called Vasculostatin-120, which inhibits endothelial cell migration, proliferation, and tube formation (Kaur et al., 2005). In Bolyard et al. (2014), the authors created an oncolytic HSV that expressed Vasculostatin-120, called 34.5ENVE. When injected intraperitoneally in a murine xenograft model of disseminated peritoneal ovarian cancer, 34.5 ENVE prolonged survival from 49 to 63 days relative to the virus control, and reduced tumor burden as measured by bioluminescence imaging. Likewise, the presence of intraperitoneal metastases and ascites at time of death was diminished with the 34.5 ENVE treatment to 25% (2/8 mice with metastasis) from 50% (4/8) with the virus control and 100% with PBS (8/8). In Meisen et al. (2015), 34.5 ENVE was then tested in the breast cancer brain metastasis (BCBM) model, where breast tumor cells Met-1 or DB-7 were injected into the brains of mice. Intratumoral injection of 34.5 ENVE decreased tumor growth and improved survival compared to untreated controls in both models. However, unlike the Bolyard et al. (2014) study, 34.5 ENVE was not compared with a control oncolytic virus in the BCBM models, and therefore it remains to be seen if expression of Vasculostatin-120 provided an important improvement to oncolytic potency.

Anti-angiogenic therapies have been promising since their discovery because angiogenesis is practically absent in normal tissues (Hanahan and Folkman, 1996; Bergers and Benjamin, 2003), so a therapy targeting it can be very specific for cancer. However, their use has not shown the expected success (Roukos et al., 2009; Ferrara and Adamis, 2016). For example, anti-angiogenic drugs such as anti-CXCL12 or Vstat120, can increase tumor hypoxia and necrosis which stimulates the secretion of pro-angiogenic factors and therefore promotes tumor growth (Potente et al., 2011). Furthermore, the abundance of pro-angiogenic factors in the tumor microenvironment favours resistance to anti-angiogenic drugs. In both cases, the dose and administration frequency will be very important (Mander and Finnie, 2018). It will be important to see if OVs that modulate angiogenic factors also come with undesirable consequences. Moreover, since CXCL12 has several functions promoting tumor growth and metastasis besides its pro-angiogenic role (Mortezaee, 2020), it is possible that inhibiting CXCL12 comes with secondary benefits for tumor reduction beyond angiogenesis reduction, and may serve as a better target.

## OV Modifications That Alter Tumor Cell Signaling

There are several factors that modulate tumor growth, invasion and metastasis, such as adhesive signals from the ECM, mechanical pressures from the ECM, cell to cell interactions, microbiome as well as soluble signals (growth factors and cytokines) (Fares et al., 2020). In the following section, we will describe different cellular signaling pathways that have been modified or exploited by OVs (summarized in Table 3) with the objective of reducing tumor growth and metastasis.

### Urokinase Plasminogen Activator and its Receptor

One of the protease systems that participates in the ECM disassembly process to promote invasion, migration and metastasis is the urokinase plasminogen activator-urokinase plasminogen activator receptor (uPA-uPAR) system (Pillay et al., 2007). uPA is a serine protease that converts plasminogen to plasmin, which participates in the degradation of fibrin, blood clotting factors and ECM (Mahmood et al., 2018). The uPA-uPAR system is overexpressed in several cancers, and its inhibition leads to tumor regression and metastasis reduction in animal models (Pillay et al., 2007). Transgenes that exploit the uPA-uPAR system have also been explored as potential boosters of oncolytic virus activity.

The authors in Morodomi et al. (2012) took advantage of the increased expression of uPA in cancer cells in designing a novel recombinant Sendai virus that had uPA-specific cell-cell fusion killing activity [rSeV/dMFct14 (uPA2) or BioKnife]. One of the modifications in this virus is that the trypsin-dependent cleavage site of the fusion (F) gene is manipulated to be susceptible to uPA and not trypsin, so that killing would be specific to uPA-expressing cells. They established an orthotopic xenograft model of human malignant mesothelioma by injecting H226-luc cells into the right thoracic cavity of nude mice. Seven days after tumor cell injection, they intrapleurally injected BioKnife-GFP or the control virus rSeV/dM-GFP. *In vivo* bioluminescence imaging demonstrated that BioKnife-GFP significantly reduced tumor burden at 7 and 14 days relative to control virus. They detected virus by GFP expression in the tumor at 7 days post-infection that correlated with increased apoptosis in the BioKnife-GFP-treated group. In Tanaka et al. (2019), BioKnife was further evaluated in a murine orthotopic head and neck squamous cell carcinoma syngeneic model where the head and neck squamous cell carcinoma (HNSCC) cell line SCCVII were injected into the floor of the mouth at day 0. Virus treatments were administered intratumorally at days 1, 2, 3 and 4. At day 4, HNSCC cells were inoculated into the subcutaneous region of the left flank to simulate metastasis. While BioKnife-GFP did not have notable effects on the primary tumor, this OV considerably reduced secondary tumor growth relative to virus control and increased CD8^+^ lymphocyte infiltration in the secondary tumor. However, more experiments are needed to totally dilucidated the role of immune cell activation in BioKnife’s mechanism.

### Transforming Growth Factor β

In the complexity of the tumor microenvironment, many molecules that participate actively in the invasion, migration and metastatic processes display complex regulatory circuits. For example, uPA/uPAR and MMPs activate the latent form of transforming factor β (TGF-β), while TGF-β regulates the expression of uPA and MMPs in cancer cells (Annes et al., 2003; Santibanez et al., 2018). TGF-β is a key cytokine in all stages of cancer development. At the beginning, it acts as tumor suppressor promoting growth arrest and apoptosis of malignant cells. Later, it functions as a tumor promoter activating cell growth, angiogenesis, EMT, metastasis, anti-tumor immune evasion and chemotherapy resistance (Hao et al., 2019). TGF-β binds and elicits its effects through TGF-β type I and type II receptors (TβRI and TβRII) that possess serine/threonine kinase activity. Several signaling pathways are activated via TGF-β, including the canonical SMAD, MAPK, RHO-like GTPase and PI3K/AKT pathway (Hao et al., 2019).

The Seth group combined the oncolytic power of adenovirus with inhibition of TGF-β signaling to generate Ad. sTβRFc, a replicating adenovirus in which the cytomegalovirus immediate early (CMV) promoter drives expression of a soluble form of TGF-β receptor II fused with human immunoglobulin Fc fragment (sTGFβRIIFc). Hu et al. (2010) tested this recombinant adenovirus in a bone metastatic xenograft tumor model established by intracardiac injection of human MDA-MB-231 breast cancer cells. Virus was injected *via* tail vein on days 4 and 7. The authors observed a significant decrease in bone metastases evaluated by X-ray and immunohistochemistry in mice treated with Ad. sTβRFc relative to virus control (Ad.luc2). Analysis of calcium levels in blood revealed reduced hypercalcemia with Ad. sTβRFc compared to virus control, indicating that the virus inhibited bone metastases and osteolytic bone destruction**.** Further studies (Hu et al., 2011) using *in vivo* imaging confirmed that Ad. sTβRFc decreased metastasis and prolonged survival relative to virus control. Ad. sTβRFc also reduced metastatic tumor burden relative to control virus in the immune competent 4T1-luc2 bone metastatic breast cancer model (Zhang et al., 2012). The efficacy of treating metastatic prostate cancer with this modified adenovirus was also tested by the Seth group (Hu et al., 2012). Bone metastases were established by intracardiac injection of nude mice with PC-3-luc cells prior to intravenous injection with Ad. sTβRFc or control virus. By whole-body bioluminescence imaging they found that Ad. sTβRFc reduced tumor growth most efficiently than virus control. Similar to results with the human breast tumor model, Ad. sTβRFc inhibited hypercalcemia and growth of prostate cancer metastases in the bone. Taking the Seth group’s publications together, inhibition of TGF-β signaling by Ad. sTβRFc seems to provide advantage in treating prostate and breast cancers that metastasize to the bone.

### Decorin

Although pro-tumorigenic signals are expected in the tumor environment, some anti-tumorigenic molecules such as decorin can be detected. Decorin belongs to the small leucine-rich proteoglycan family of proteins and is a component of the ECM (Sofeu Feugaing et al., 2013). In the matrix, decorin acts as anti-tumorigenic agent by repressing signal transduction pathways such as cell proliferation, angiogenesis, and migration (Neill et al., 2012; Sofeu Feugaing et al., 2013; Zhang et al., 2018).

The Seth group, with the focus on manipulating signaling pathways involved in cancer progression, created an adenovirus expressing human decorin (Ad.dcn) (Xu et al., 2015). The authors evaluated the activity of Ad. dcn in the same xenograft metastatic PC-3-luc prostate cancer mouse model used in Hu et al. (2012) using *in vivo* bioluminescence, X-ray, and micro-computed tomography to monitor tumor burden. They observed that Ad. dcn significantly inhibited tumor progression, decreased bone destruction and prolonged survival relative to the virus control without decorin (Ad.luc). Similarly, Yang et al. (2015) showed that Ad. dcn inhibited growth of bone metastases in a xenograft MDA-MB-231 breast cancer mouse model. More recently, Zhao et al. (2019) explored the ability of Ad. dcn to inhibit pulmonary metastasis in the highly aggressive syngeneic 4T1-luc orthotopic mouse model. When mammary tumors were palpable (∼7 days) and at day 10, viruses were injected either intratumorally or intravenously. Lung metastases were analyzed at day 25 by histopathological assays. They determined that intratumoral or intravenous deliveries of Ad. dcn reduced tumor growth and pulmonary metastases, increasing the frequency of lung metastasis-free-mice relative to virus control (Ad.Null). However, intratumoral injections were more effective at reducing primary tumor growth and expressing the transgene, whereas intravenous delivery was more successful at preventing lung metastases. Decorin target genes were downregulated in the tumor as well as in the metastases, indicating a direct activity of virus-derived decorin on cell signalling. It would be interesting to establish if decorin activities at metastases are from direct virus replication at metastatic sites or via circulation. Should decorin (or any virus-derived cytokine) be found in circulation, it would be necessary to ensure that the levels do not negatively affect healthy tissues. Furthermore, Ad. dcn treatment, systemically or intratumorally, induced an upregulation of CD8^+^ T cells in peripheral blood.

To boost the innate immune response, decorin was combined with granulocyte macrophage colony stimulating factor (GM-CSF), an immune stimulator of natural killer cells, macrophages, and dendritic cells in an oncolytic adenovirus (Liu et al., 2017). In this virus (rAd.DCN.GM), decorin was expressed under control of the CMV immediate early promoter, while GM-CSF expression was driven by the E1B promoter. Cancer-specific virus replication was controlled by placing the TERT promoter upstream of E1A. Using the CT26 xenograft model of colorectal cancer, the authors demonstrated that intratumoral injection of virus (rAd.DCN, rAd.GM, or rAd.DCN.GM) significantly decreased tumor volume relative to treatment with rAd.Null and mock. When pulmonary metastases were analyzed, 5/6 mice were tumor-free in the rAd.DCN.GM group, 4/6 mice were tumor-free in the rAd.GM and rAd.DCN groups, while only 2/6 mice were tumor-free in the rAd.Null-treated group. rAd.DCN.GM increased CD8^+^ T cells in spleen and peripheral blood, reduced TGF-β expression and augmented dendritic cells in the spleen, suggesting that both decorin and GM-CSF contribute to rAd.DCN.GM mechanisms of action.

### TNF-α/TRAIL

Programmed cell death or apoptosis can be beneficial for oncolytic therapy because it does not only kill the tumor cell but also releases tumor antigens that stimulate the anti-tumor immune response (Zhou et al., 2019b). Tumor necrosis factor alpha (TNFα) is a cytokine produced by immune cells such as macrophages and monocytes that, besides its role inducing apoptosis and necrosis, can regulate inflammation, growth, and proliferation of normal and transformed tissues (Atzeni and Sarzi-Puttini, 2013; Fitzgerald et al., 2013). The localized production of this cytokine by oncolytic viruses can be very beneficial by preventing systemic toxicity. Consequently, TNFα coding sequences have been added to an oncolytic adenovirus with an Ad5/3 chimeric capsid and a 24 bp deletion in the constant region 2 of E1A to make virus replication selective for tumor cells with a defective retinoblastoma/p16 pathway. Armed virus Ad5/3-D24-hTNFa produced TNFα in tumors, reduced tumor growth and improved survival relative to control virus in a PC-3 MM2 xenograft murine model of prostate cancer. It also reduced tumor growth and increased tumor specific CD8 T cells in a metastatic B16-OVA immunocompetent murine model of melanoma (Hirvinen et al., 2015), although it should be noted that human adenovirus does not replicate in murine cells. Later, the same group tested the armed virus in combination with another anti-tumor inflammatory cytokine IL-2 (Havunen et al., 2017). The new armed virus Ad5/3-E2F-d24-hTNFa-IRES-hIL2 (or OAd.TNFa-IL-2) with the transgenes incorporated into the E3 region, showed a significant reduction in tumor growth in an HapT1 immunocompetent Syrian hamster model relative to control unarmed virus (OAd). OAd.TNFa-IL-2 virus also increased CD4/CD8 T cell infiltration in the tumor microenvironment (Havunen et al., 2017).

Three research groups have introduced TNF-related apoptosis-inducing ligand (TRAIL) to adenovirus OVs to increase apoptosis of tumoral cells. In its native form TRAIL is a transmembrane protein that binds to death receptors DR4 and DR5 to induce extrinsic apoptosis (Yuan et al., 2018), although most therapeutic agents incorporating TRAIL use an engineered soluble form of the protein. Soluble TRAIL has the advantage of acting on uninfected tumor cells near the site of injection. In Sova et al. (2004), the authors created an Ad5/35 fiber-substituted oncolytic adenovirus that infects cells independently of the coxsackievirus and adenovirus receptor (CAR) and instead enters via CD46 which is highly expressed in malignant tumor cells. The TRAIL transgene was inserted into this virus, creating the oncolytic vector Ad. IR-E1A/TRAIL. The viruses were tested in the xenograft model of liver metastasis generated by infusing human LoVo colorectal carcinoma cells via the portal vein into immunodeficient CB17 mice. Two weeks after two sequential intravenous injections of virus, mice were euthanized and evaluated for liver metastases. The authors found that administration of Ad. IR-E1A/TRAIL reduced tumor burden 10-fold relative to untreated mice and approximately 2-fold relative to the virus control Ad. IR-E1A/AP (without TRAIL), without causing toxicity to the liver.

In an independent effort to apply TRAIL towards enhancing adenovirus-based OV potency, Zhu et al. (2013) combined two modifications of the E1 region of adenovirus to increase the specificity of virus replication to tumor cells: placing adenoviral genes E1A and E1B under the control of hTERT promoter and HRE (hypoxia response element) respectively. Into this viral backbone they incorporated a TRAIL expression cassette driven by the CMV promoter, creating P55-HTERT-HRE-TRAIL. This TRAIL-expressing virus decreased tumor growth in a mouse xenograft orthotopic model of breast cancer (MDA-MB-213 cells). Higher levels of apoptosis measured as TUNEL staining were found in P55-HTERT-HRE-TRAIL treated tumors versus virus control (P55-HTERT-HRE). When the virus activity was evaluated in a simulated model of metastasis (MDA-MB-231-luc injected into the left heart ventricle), P55-HTERT-HRE-TRAIL-treated group showed 60% survival at day 60 whereas the virus control group (P55-HTERT-HRE) showed only 20% survival. The reduction of metastases was confirmed with *in vivo* imaging every 7 days.

Lastly, Zhou et al. (2017) developed an adenovirus expressing TRAIL and viral E1A under control of the tumor-specific hTERT promoter. They evaluated the effect of intraperitoneal injection of Ad/TRAIL-E1 on metastasis in an *in vivo* MKN45 cell peritoneal carcinomatosis xenograft mouse model. They found that Ad/TRAIL-E1 significantly reduced the number of mesentery tumors (22.8 ± 10.3) relative to the virus control (Ad/GFP-E1, 65.3 ± 34.4). Ad/TRAIL-E1 also reduced tumor weight and increased survival relative to Ad/GFP-E1 although the difference was not statistically significant. The expression of TRAIL and the level of apoptosis in the disseminated tumors was higher in the Ad/TRAIL-E1-treated group than in the virus control group.

### Gene Expression Regulators: STAT3 and SATB1

The examples of genetic modifications of OVs discussed above focus on blocking ligand-receptor interactions. Other groups have modified OVs to targeted molecules that are downstream of receptors in various signaling pathways. One such target is the transcription factor STAT3 which is downstream of cytokine and growth factor receptors. This factor is involved in the regulation of autonomous properties of tumor cells such as proliferation as well as communication with other cells in the tumor microenvironment, resulting in increased vascularization, migration, invasion, and immunosuppression (Groner et al., 2008). Han et al. (2009) modified an oncolytic adenovirus to inhibit STAT3 by inserting a 770 bp antisense fragment of STAT3 into the ADP locus of Ad5/dE1A, previously generated with a deletion of amino acids 121–129 in E1A. They evaluated the resulting virus, M4, for its ability to inhibit metastasis in an orthotopic model of gastric cancer established using explanted MKN-45 xenograft tumor fragments (Huang et al., 2008). Viruses were injected into the tail vein for five consecutive days, then 6 weeks later mice were assessed for tumor growth and metastases. The authors observed that M4 prolonged survival and decreased tumor growth, invasion of the liver, and peritoneal dissemination compared to control virus without the STAT3 antisense sequence. Importantly, M4 also decreased STAT3 expression in tumors. As STAT3 is involved in immunosuppression, it would be interesting to examine the activity of this virus in an immune competent mouse model as well.

Another key regulator of tumor progression and metastasis is the transcription factor SATB1. SATB1 belongs to the SATB (Special AT-rich Binding protein) family. These proteins are high-order chromatin organizers, and histone and post-translational modifiers (Naik and Galande, 2019). STAB1 is highly expressed in numerous malignancies, including breast, prostate, liver, and bladder cancers. In addition, SATB1 promotes a highly aggressive phenotype due to its role activating the EMT process that leads to metastasis and invasion (Glatzel-Plucinska et al., 2019). In order to silence this important tumorigenic factor, Mao et al. (2015) constructed the virus ZD55-SATB1, in which the E1B-55K sequence was replaced with a SATB1-targeted shRNA expression cassette. The authors evaluated ZD55-SATB1 in the subcutaneous DU145 prostate cancer model. ZD55-SATB1 inhibited growth of primary tumors and lung micrometastases. Histopathological analyses of tumors revealed that ZD55-SATB1 inhibited expression of SATB1 and induced a higher level of apoptosis than the virus control (ZD55-EGFP).

### Beclin-1

Cell death can be the result not only of apoptosis, but also of other processes such as autophagy. The induction of autophagy has been explored by some groups introducing Beclin-1 to VV. Beclin-1’s phosphorylation regulates the initiation of autophagy, facilitating the recruitment of autophagic proteins and autophagosome biogenesis (Menon and Dhamija, 2018). The OVV-BECN1 was created in a VV backbone with a TK viral gene deletion for tumor selectivity. OVV-BECN1 induced cell death through autophagy and not apoptosis in hematologic malignant cells *in vitro*. On the other side, OVV-BECN1 reduced tumor growth and increased survival significantly in a K62-luciferase cells xenograft murine model of leukemia. Presence of Beclin-1 and autophagic vacuoles were found in the OVV-BECN1 treated tumors by IHQ and electron microscopy respectively (Lei et al., 2020). OVV-BECN1 also decreased tumor growth in a murine non-Hodgkin lymphoma xenograft model (Xie et al., 2021).

### CD147

Various molecules are upregulated on the surface of tumor cells to support cancer progression, including CD147, a glycoprotein involved in regulation of the tumor microenvironment and tumor growth. CD147 induces the expression of MMPs and the uPA/uPAR system promoting invasion and metastasis. In addition, CD147 regulates tumor cell adhesion and angiogenesis (Iacono et al., 2007; Landras et al., 2019). Strategies have been developed to block CD147 activity because of its important role in cancer progression (Iacono et al., 2007; Landras et al., 2019). Wei et al. (2015) used reverse genetics to construct a recombinant Newcastle disease virus (NDV) expressing an antibody against CD147 (rNDV-18HL). They tested rNDV-18HL in the SMMC-7721 orthotopic hepatoma model. Starting 1 week after implantation, viruses were intravenously injected twice weekly for 3 weeks. Virus replication and anti-CD147 antibody were detected at the tumor site by immunohistochemistry. Furthermore, mice treated with rNDV-18HL showed a significantly reduced number of intrahepatic metastases and prolonged survival relative to virus control (NDV Italien). Future studies may demonstrate the utility of this novel approach using oncolytic viruses delivering therapeutic antibodies to the tumor site.

### Tumor Suppressor TSLC1

Adhesion proteins and other molecules are important to maintain tissue structure and organization. In some cases, downregulation of these molecules in the tumor microenvironment can promote invasion and metastasis. This is the case of the tumor suppressor lung 1 (TSLC1) protein, a cell-cell adhesion protein that also functions intracellularly by interacting with several signaling proteins involved in tumorigenesis, supressing EMT and inducing apoptosis (Liang et al., 2011). The Wang group (Zhang et al., 2017) investigated whether oncolytic adenovirus delivery of TSLC1 specifically to cancer stem cells (CSCs) of hepatocellular carcinoma (HCC) could impact tumor progression. To do this, they created an adenovirus that encodes TSLC1 and placed expression of the viral delta-24 E1A protein (unable to bind Rb) under the control of Wnt promoter (Ad.wnt-E1A (Δ24bp)-TSLC1). Wnt signaling is highly activated in CSC supporting self-renewal ability and multi-differentiation potential (de Sousa E Melo and Vermeulen, 2016). To test Ad. wnt-E1A (delta24bp)-TSLC1 and its efficacy targeting CSCs *in vivo*, they established a tumor model by injecting subcutaneously MHCC-97H-luc spheres. When tumors reached 100 mm^3^, test and control viruses were injected intratumorally. Tumor growth was monitored *in vivo* by bioluminescence imaging and showed a significant reduction in tumor burden in mice treated with Ad. wnt-E1A (Δ24bp)-TSLC1 compared to mice treated with the control (Ad.wnt-E1A (Δ24bp)-EGFP). In addition, the number of metastatic nodules were significantly reduced in Ad. wnt-E1A (Δ24bp)-TSLC1-treated mice. This study demonstrated that CSCs can be effectively targeted by oncolytic adenovirus, and that overexpression of the tumor suppressor TSLC1 may reduce metastasis.

Overall, this section describes that many of the signalling pathways involved in tumor invasion and metastasis processes are possible candidates for manipulation through oncolytic viruses that deliver exogenous genes to the tumors. So far, OV researchers have focused on manipulating signalling pathways that modulate the ECM and tumor microenvironment. Studies to date have concentrated on evaluating the overall change to primary tumor and metastatic burden, without in-depth analysis of relative virus burden and spread, or specific changes to tumor and tumor-supporting cells or immune cell populations. In future, approaches such as single-cell sequencing of tumor and metastatic samples could contribute immensely towards establishing the best ways to apply these modified OVs. For example, while blocking CD147 lead to reduced metastasis, it also reduced MMP expression which may inadvertently dampen dissemination of the OV as described in section B. Determining What then is the best balance of inhibiting CD147 versus encouraging MMP activity? It will be interesting to test the effect of re-introducing MMPs to CD147-inhibited conditions, to establish if virus dissemination is restored and survival further enhanced.

## Conclusion

**FIGURE 1:**
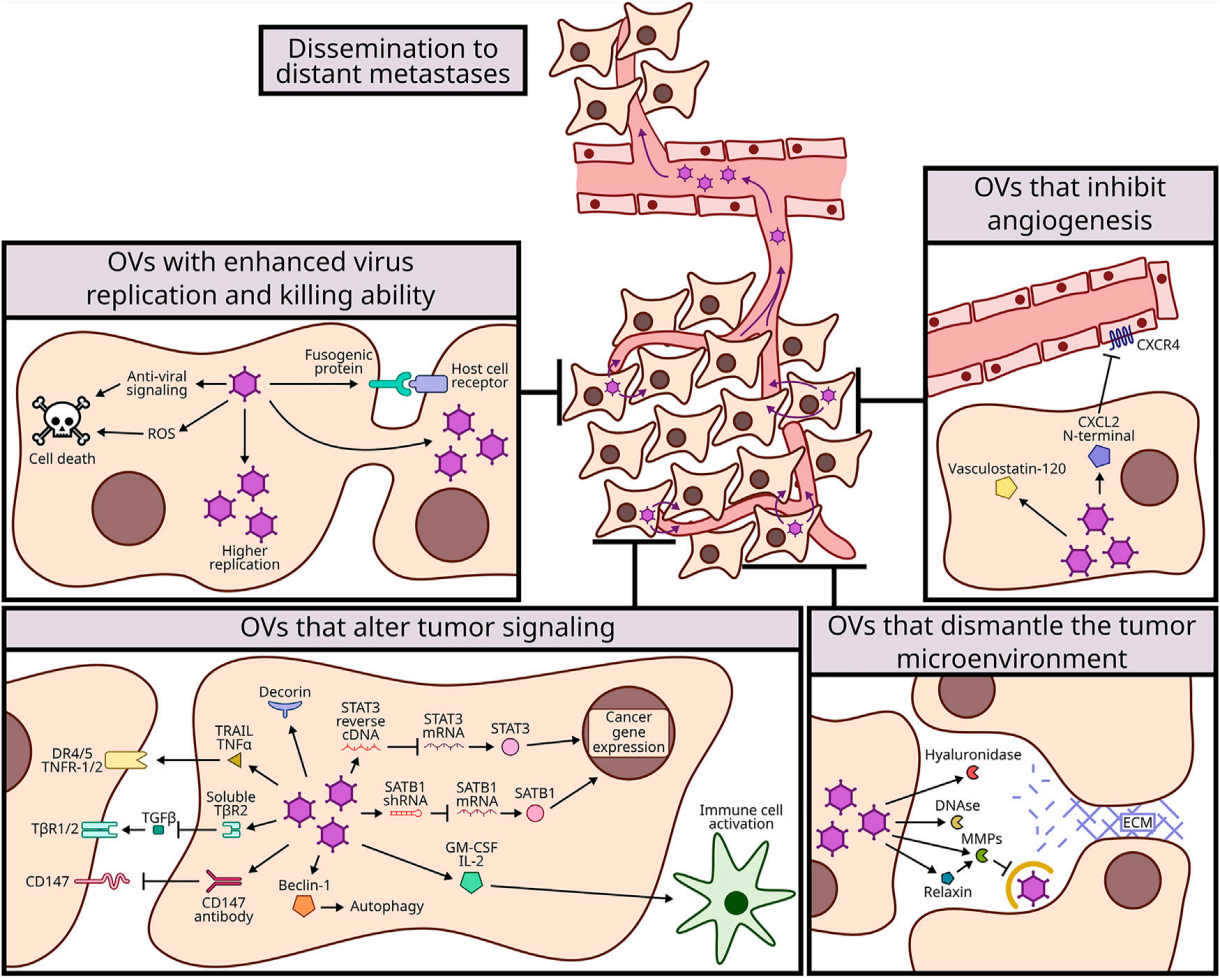
Mechanisms of genetic modifications to improve oncolytic viruses (OVs) dissemination to distant metastases. OVs have been modified to expand their oncolytic potency and with that to spread more efficiently to metastatic sites. These modifications were categorized in 4 mechanisms: enhancing virus replication and killing ability (top left, section A and Table 1); dismantling the tumor microenvironment (bottom right, section B and Table 2); inhibiting angiogenesis (top right, section C and Table 3); and altering tumor signaling (bottom left, section D and Table 3).

We have summarized and contextualized many approaches to genetically modify OVs to either support improved virus replication and spread, or to help dismantle the tumor microenvironment. The modifications described in this review were all able to improve oncolytic therapy, either by reducing primary tumor growth or metastasis. We have broadly categorized the advancements into those that (A) promote virus replication in tumors and/or death of tumor cells, (B) overcome the ECM barrier to virus dissemination within tumors or to metastatic sites, (C) reduce angiogenesis, and (D) stimulate cell signalling pathways to dismantle the tumor microenvironment or promote cell death (Figure 1). There have also been many genetic modifications to OVs aimed specifically at enhancing anti-tumor immunity, but these are already aptly described in complementary reviews (de Graaf et al., 2018; Jamieson et al., 2020; Zhang et al., 2020).

Remaining Challenges: While writing this review, we have noticed several general limitations that if overcome, could help further advance oncolytic viruses. First, most modifications to OVs have focused on DNA viruses such as adenovirus, HSV and vaccinia virus. The focus on DNA viruses is likely because these viruses have a large genome size and are more-easily manipulated. However, given that reverse genetics approaches for RNA viruses are rapidly advancing, it would be interesting to test some of the modifications described in this review in RNA viruses that possess oncolytic activity but lack sufficient oncolytic potency. For example, VSV, measles virus, NDV, coxsackieviruses and polioviruses are all being develop into oncolytic viruses and may benefit from some of the genetic modifications summarized in this review. Second, unfortunately some publications did not compare the modified virus with the control unmodified virus, making it difficult to determine the benefit of the specific genetic alteration. Third, each publication uses its own animal model, and therefore it is challenging to compare between models, OVs, and other standard therapies. It would be worthy to standardize and compare the best therapies in the same models with uniform protocols. Fourth, most studies focused on measurements of tumor size and metastatic burden, leaving many molecular insights unknown. For example, it was not always clear if the genetic modification of the virus functioned as anticipated to manipulate the desired molecular pathway or process. It was also not always clear the effect of modifications on virus amplification, tumor cell death, or anti-tumor immunity. In future, delineating the molecular details of the oncolytic viruses will allow best advancements to overcome remaining deficiencies in activities. Lastly, clinical testing is needed to fully evaluate the OVs described in this review, since responses of mice do not always predict responses in humans.

Hope for future: Although we have categorized the OV genetic modifications according to their dominant activity**,** the modifications are probably interconnected; for example, a modification that makes the OV more efficient at tumor cell killing is likely also to expose more tumor antigens and increase the anti-tumor immune response. As another example, modifying the CXCL12/CXCR4 signaling pathway to alter angiogenesis will also likely attract more immune cells that respond to this chemokine. As methods such as single-cell sequencing become more affordable, it will be very exciting to achieve a more wholistic view of the effects of each genetic modification to OVs.

When considering that each individual change described in this review made at least an incremental improvement to the activity of the oncolytic virus, it is very likely that combination of modifications could achieve the potency needed for durable cancer therapy. The trick will be to fully understand the mechanisms of each approach and the impact on virus, tumor, and immunity, so that combinations of genetic modifications have additive or ideally synergistic effects. If then considering that most modifications improved T cell infiltration, addition of checkpoint inhibitors to overcome immune suppression could further promote tumor-specific immunity. Ultimately, the optimal combination of genetically modified OVs, other cancer-targeting drugs, and tumor immunity-stimulating therapies will be achieved.
